# Ultrasound guided aspiration of hydrosalpinx fluid versus salpingectomy in the management of patients with ultrasound visible hydrosalpinx undergoing IVF-ET: a randomized controlled trial

**DOI:** 10.1186/s12905-015-0177-2

**Published:** 2015-02-27

**Authors:** Usama M Fouda, Ahmed M Sayed, Hatem I Abdelmoty, Khaled A Elsetohy

**Affiliations:** Department of Obstetrics and Gynecology, Faculty of Medicine, Cairo University, Cairo, Egypt

**Keywords:** Hydrosalpinx, Salpingectomy, IVF-ET, Infertility, Ultrasound

## Abstract

**Background:**

The aim of this study was to compare the efficacy of ultrasound guided aspiration of hydrosalpinx fluid at the time of oocyte retrieval with salpingectomy in the management of patients with ultrasound visible hydrosalpinx undergoing IVF-ET.

**Methods:**

One hundred and sixty patients with ultrasound visible hydrosalpinx were randomized into salpingectomy group and aspiration group using computer generated randomization list and sequentially numbered sealed envelopes containing allocation information written on a card.

**Results:**

The clinical pregnancy rate per started cycle and the implantation rate were non- significantly higher in the salpingectomy group compared with the aspiration group [40% vs. 27.5% (*p* value = 0.132) and 18.95% vs. 12.82% (*p* value =0.124), respectively]. In the aspiration group, 34.21% of patients had rapid re-accumulation of the hydrosalpinx fluid (i.e. within first two weeks after embryo transfer). Whereas, the clinical pregnancy rate per transfer cycle and the implantation rate were significantly higher in salpingectomy group compared with the subgroup of patients with rapid re-accumulation of hydrosalpinx fluid [42.67% vs. 19.23% (*p* value = 0.036) and 18.95% vs. 7.58% (*p* value = 0.032), respectively], no significant differences were detected between the salpingectomy group and the subgroup of patients with no re-accumulation of hydrosalpinx fluid (42.67% vs. 34% (*p* value = 0.356) and 18.95% vs. 15.5% (*p* value = 0.457), respectively).

**Conclusion:**

The small sample size could be the cause of failure of detecting significant increase in implantation and pregnancy rates in salpingectomy group compared with aspiration group. Further larger randomized controlled trials are needed to determine whether salpingectomy is more effective than aspiration of hydrosalpinx fluid or not. Moreover, the data presented in this study suggested that rapid re-accumulation of hydrosalpinx fluid is an obstacle against successful implantation and the cause of lower success rate with ultrasound guided aspiration of hydrosalpinx fluid compared with salpingectomy.

**Trial registration:**

Clinical trials.gov (NCT02008240), registered 8 December 2013.

## Background

The association of hydrosalpinx with decreased pregnancy and implantation rates in IVF cycles has been confirmed by overwhelming scientific evidence [1,2]. It has been suggested that the retrograde spillage of hydrosalpinx fluid into the uterine cavity could adversely affect embryo development, reduces endometrial receptivity by decreasing the expression of endometrial receptivity markers (HOXA10, β-integrin and leukemia inhibitory factor), prevents the contact of embryos with endometrial surface or simply wash-out the embryos [3,4].

In 1994, a retrospective study revealed that patients with hydrosalpinx undergoing IVF-ET had lower pregnancy rates compared with patients with hydrosalpinx who underwent salpingectomy prior to IVF-ET [5]. Subsequent retrospective and prospective randomized studies confirmed that salpingectomy or proximal tubal occlusion improves the outcomes of IVF-ET [6-8]. In 2010, a meta-analysis of various prospective randomized studies revealed that laparoscopic salpingectomy or proximal tubal occlusion increases the odds of clinical pregnancy, ongoing pregnancy and live birth [9]. However, salpingectomy or proximal tubal occlusion requires hospitalization, general anesthesia and may be associated with operative complications particularly in patients with dense adhesions. Moreover, bilateral salpingectomy precludes any possibility of future unassisted conception or tubal repair [6,7].

Several management options as hysteroscopic tubal occlusion by Essure micro-inserts, ultrasound guided aspiration of hydrosalpinx and medical treatment (antibiotics and/or corticosteroids) were suggested as alternatives to salpingectomy or proximal tubal occlusion. Several authors suggest that ultrasound guided aspiration of hydrosalpinx fluid is the best alternative because it is simple, safe, easy and inexpensive. Furthermore, the evidence supporting its beneficial effect on the outcomes of IVF-ET comes from several prospective randomized controlled studies [10,11]. On the other hand, the literature on Essure micro-inserts and antibiotics treatment was limited to small retrospective studies or prospective non-randomized studies [12,13]. Moreover, the occlusion of fallopian tube with Essure micro-inserts is expensive, delays IVF-ET cycle for 3 months and its risk to the patients who become pregnant and their fetuses is not known [14].

The aim of this randomized controlled trial was to compare the efficacy of ultrasound guided aspiration of hydrosalpinx fluid with salpingectomy in the management of patients with ultrasound visible hydrosalpinx undergoing IVF-ET.

## Methods

This prospective, two arm, allocation concealed, randomized controlled trial was conducted at the assisted conception unit of Aljazeera (Al Gazeera) hospital, Giza, Egypt, during the period between July 2011 and May 2014. The study included 160 patients with unilateral or bilateral hydrosalpinx visible by ultrasound. Exclusion criteria were age more than 37 years or less than 18 years, FSH ≥ 12IU/L, uterine fibroids requiring surgical removal, irregular cycles, previous IVF cycles, body mass index less than 19 or more than 30, endometriosis, habitual abortion or systemic disease contraindicating pregnancy. The study protocol was approved by the institutional review board of Aljazeera (Al Gazeera) hospital. The patients were counseled about the benefits and risks of both management options and written informed consent was obtained from all patients. The study was open labeled due to the surgical nature of treatment.

The patients were randomly allocated to salpingectomy group or aspiration group (1: 1 ratio) using computer generated randomization list and sequentially numbered sealed envelopes containing allocation information written on a card. The sealed envelopes were prepared by statistician not involved in the study. Study nurse opened the sequentially numbered envelopes to allocate patients to the assigned group.

In laparoscopic salpingectomy group, the mesosalpinx was coagulated as close as possible to the fallopian tube using bipolar electrocoagulation to avoid any compromise in the ovarian blood supply. Bilateral salpingectomy was performed in patients with bilateral hydrosalpinx. Proximal tubal occlusion and distal fenestration of hydrosalpinx were done in cases with extensive pelvic adhesions. A minimum period of 2 months between surgery and oocyte retrieval was recommended.

GnRH agonist triptorelin (Decapeptyl, Ipsen,Slough,United Kingdom) was started one week before anticipated menstruation at a dose of 0.1 mg/day. On day 3 of cycle, pituitary down regulation was confirmed (serum E2 levels less than 50 pg/ml and endometrial thickness less than 5 mm) and highly purified urinary FSH (HP-uFSH) (Fostimon, IBSA, Switzerland) was started. The starting dose of HP-uFSH ranged between 150 IU to 300 IU (depending on ovarian reserve indicators as age, antral follicle count and basal FSH). The daily dose of HP-uFSH was adjusted 5 days after starting stimulation depending on follicular development as observed by ultrasonography and serum estradiol. Triptorelin and HP-uFSH were continued up to and including the day of human chorionic gonadotropin (HCG) (Pregnyl; N.V. Organon, Oss, Holland) administration. Ovulation was triggered by 10000 IU HCG when 3 or more follicles (18 mm or more in diameter) were detected by ultrasound examination. Transvaginal ultrasound guided oocyte retrieval was done under deep sedation 34 to 36 hours after HCG administration. Both groups received Azithromycin 1000 mg orally and 1 g Cefotaxime I.M before oocyte retrieval.

In aspiration group, an aspiration needle was inserted into the hydrosalpinx after oocyte retrieval and suction was applied to aspirate the hydrosalpinx fluid completely. In cases with bilateral hydrosalpinx, the process was repeated on the opposite side. The aspirated hydrosalpinx fluid was sent for microbiological examination.

A maximum of 3 embryos were transferred per patient 2 or 3 days after oocyte retrieval. Serum HCG test was done 14 days after embryo transfer to detect pregnancy and ultrasound examination was done 5 weeks after embryo transfer to confirm fetal viable and to detect the number of sacs in the uterus. Ectopic pregnancy was considered an implanted embryo. Luteal phase was supported with progesterone suppositories 200 mg twice daily (Prontogest, Marcyrl Pharmaceutical Industries, El Obour, Egypt) starting from the day of oocyte retrieval till 12 weeks pregnancy or negative pregnancy test.

In both groups, the presence of uterine fluid collection was assessed during ultrasound guided oocyte retrieval and during ultrasound guided embryo transfer. In aspiration group, transvaginal ultrasound examinations were performed on the day of embryo transfer and 2 weeks after embryo transfer to detect the re-accumulation of hydrosalpinx fluid.

The primary outcome measure was the clinical pregnancy rate per started cycle (presence of intrauterine gestation sac detected by transvaginal ultrasound) and the secondary outcome measures were ongoing pregnancy rate (pregnancies continued beyond 20 weeks gestation), implantation rate, abortion rate, ectopic pregnancy rate, operative complications and flaring of pelvic infection.

### Sample size calculation

This study aimed to reveal non-inferiority of ultrasound guided aspiration of hydrosalpinx versus salpingectomy in the management of patients with ultrasound visible hydrosalpinx due to undergo IVF-ET. A recent randomized controlled trial revealed that the clinical pregnancy rate in patients with ultrasound visible hydrosalpinx was 13.21% and a recent meta-analysis revealed that the clinical pregnancy rate in patients with hydrosalpinx who underwent salpingectomy prior to IVF-ET was 36.73% [11,9]. If we use a difference in the clinical pregnancy rate between the experimental (aspiration of hydrosalpinx fluid) and the conventional strategy (salpingectomy) of – 5% as the critical threshold for non-inferiority. At least 2300 patients (1150 in each arm) are required to be 80% sure that the upper limit of a one-sided 95% confidence interval will exclude a difference in favor of the salpingectomy group of more than 5%, if there is truly no difference between the salpingectomy and aspiration of hydrosalpinx in clinical pregnancy rate. This was not feasible in a study which was planned to be finished within 3 years. Therefore, we choose on arbitrary bases to include 80 patients in each arm of the study. The main purpose of the study was to provide data comparing the efficacy of ultrasound guided aspiration of hydrosalpinx fluid with salpingectomy in the management of patient with hydrosalpinx due to undergo IVF-ET, this data could be used in future meta-analyses comparing both management options.

### Statistical analysis

Statistical analysis was performed using an intention-to-treat analysis and per protocol analysis. Data were analyzed by statistical package for the social science software. Student t and Fisher exact tests were used as appropriate. A *p* value less than 0.05 was considered statistically significant.

## Results

**Figure 1:**
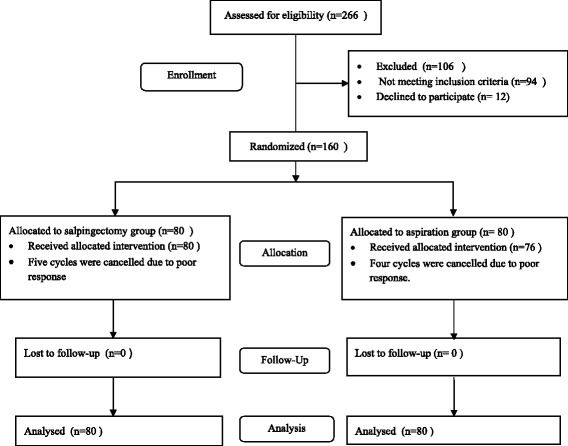
**Flow of the patients in the study.**

Two hundred and sixty six patients with ultrasound visible hydrosalpinx were assessed for eligibility to this study. Ninety four patients did not meet the inclusion criteria and 12 patients refused to participate in the study. Thus, 160 patients were recruited to the study. Eighty patients were randomized to each group. The flow of the patients in the study is shown in Figure 1.

Table 1 shows the demographic criteria of the patients. There were no significant differences between both groups with respect to age, body mass index, basal FSH, duration of infertility and percentage of patients with bilateral hydrosalpinx.

**Table 1:** **Patients’ characteristics**

	**Salpingectomy group (n = 80)**	**Aspiration group (n = 80)**	**P value**
**Age (years)**	**28.14 ± 3.67**	**27.55 ± 3.52**	**0.303**
**Body mass index (Kg/m** ^**2**^ **)**	**25.7 ± 2.06**	**25.3 ± 1.92**	**0.205**
**Duration of infertility (years)**	**3.66 ± 1.86**	**3.29 ± 1.78**	**0.194**
**Basal FSH (IU/L)**	**6.32 ± 2.05**	**6.08 ± 1.98**	**0.446**
**Bilateral hydrosalpinx**	**16/80 (20%)**	**20/80 (25%)**	**0.571**

Values are expressed as mean ± SD or n/n (%).

Table 2 shows the cycle characteristics in both groups. The stimulation period, total dose of HP-uFSH, number of follicles ≥ 18 mm on the day of HCG administration, oocytes retrieved, metaphase II oocyte, 2 pro-nucleate (2PN) embryos and number of embryos transferred were comparable between both group.

**Table 2:** **IVF cycle characteristics**

	**Salpingectomy group (n = 80)**	**Aspiration group (n = 80)**	***P*** **value**
**Stimulation period (days)**	**11.4 ± 1.13**	**11.68 ± 1.52**	**0.197**
**Consumed HP-uFSH units**	**2752.5 ± 664.5**	**2869.5 ± 681.75**	**0.272**
**Follicles ≥ 18 mm on the day of HCG administration**	**10.93 ± 4.35**	**11.33 ± 4.76**	**0.580**
**Retrieved oocytes**	**10.53 ± 3.50**	**10.87 ± 4.17**	**0.593**
**Metaphase II oocytes**	**9.17 ± 3.54**	**9.70 ± 3.98**	**0.394**
**Two pronucleate embryos**	**7.35 ± 3.22**	**7.70 ± 3.55**	**0.526**
**Fertilization rate**	**551/688 (80.09%)**	**585/737 (79.38%)**	**0.742**
**No. of embryos transferred**	**2.53 ± 0.5**	**2.57 ± 0.49**	**0.691**
**Grade I & II embryos /transferred embryos**	**146/190 (76.84%)**	**153/195 (78.46%)**	**0.715**

Values are expressed as mean ± SD or n/n (%).

Proximal tubal occlusion was done in 4 patients due the presence of extensive pelvic adhesions. Cultures of hydrosalpinx fluid were negative. There was no flaring of pelvic infection or peritonitis in the aspiration group or surgical complications in the salpingectomy group.

The implantation and pregnancy rates per started cycle (intention to treat analysis) and per transfer cycle (per protocol analysis) were higher in the salpingectomy group compared with the aspiration group but these differences failed to reach statistical significance. The abortion rate was comparable between both groups. A case of tubal ectopic pregnancy was reported in the aspiration group (Table 3).

**Table 3:** **Reproductive outcomes**

	**Salpingectomy group (n = 80)**	**Aspiration group (n = 80)**	**Odd ratio (95% CI)**	**P value**
**No. of transfer cycles**	**75**	**76**		
**Clinical pregnancy/started cycle**	**32/80 (40%)**	**22/80 (27.5%)**	**1.76 (0.9,3.41)**	**0.132**
**Ongoing pregnancy/started cycle**	**29/80 (36.25%)**	**19/80 (23.75%)**	**1.83 (0.92,3.63)**	**0.120**
**Clinical pregnancy rate /transfer cycle**	**32/75 (42.67%)**	**22/76 (28.95%)**	**1.83 (0.93,3.59)**	**0.091**
**Ongoing pregnancy rate /transfer cycle**	**29/75 (38.67%)**	**19/76 (25%)**	**1.89 (0.94,3.8)**	**0.082**
**Implantation rate**	**36/190 (18.95%)**	**25/195 (12.82%)**	**1.59 (0.91,2.77)**	**0.124**
**Spontaneous abortion rate n/IUP**	**3/32 (9.38%)**	**3/22 (13.64%)**	**0.66 (0.12, 3.59)**	**0.678**

Values are expressed as n/n (%) unless otherwise indicated. IUP = intrauterine pregnancy.

In the aspiration group, ultrasound examination on the day of embryo transfer revealed that 3 patients had uterine fluid collection and re-accumulation of hydrosalpinx fluid and 8 patients had re-accumulation of hydrosalpinx fluid. When the patients were re-scanned 2 weeks after embryo transfer, re-accumulation of hydrosalpinx fluid was detected in 26 patients. No significant differences in age, body mass index, basal FSH, duration of infertility and percentage of patients with bilateral hydrosalpinx were detected between the subgroup of patients with rapid re-accumulation of hydrosalpinx fluid (i.e. within first 2 weeks after embryo transfer) and the subgroup of patients with no re-accumulation of hydrosalpinx fluid (Table 4). None of the patients in the salpingectomy group had uterine fluid collection detected by ultrasound.

**Table 4:** **Patients’ characteristics and reproductive outcomes per transfer cycle in the salpingectomy group and the subgroups of the aspiration group**

	**Salpingectomy (Group 1)**	**No re-accumulation of hydrosalpinx fluid (Group 2)**	**Re-accumulation of hydrosalpinx fluid (Group 3)**	**G1 Vs G2**	**G1 Vs G3**	**G2 Vs G3**
**No. of transfer cycles**	**75**	**50**	**26**			
**Age**	**27.92 ± 3.59**	**28.2 ± 3.63**	**27.04 ± 3.01**	**0.672**	**0.228**	**0.143**
**Body mass index (Kg/m** ^**2**^ **)**	**25.68 ± 2.03**	**25.36 ± 1.96**	**25.19 ± 1.81**	**0.379**	**0.257**	**0.711**
**Bilateral hydrosalpinx**	**15/75 (20%)**	**12/50 (24%)**	**7/26 (26.92%)**	**0.660**	**0.582**	**0.786**
**Duration of infertility**	**3.56 ± 1.8**	**3.26 ± 1.69**	**3.54 ± 2**	**0.346**	**0.962**	**0.548**
**Basal FSH (IU/L)**	**6.35 ± 2.09**	**6.29 ± 2.17**	**5.81 ± 1.68**	**0.872**	**0.188**	**0.288**
**No. of embryos transferred**	**2.53 ± 0.5**	**2.58 ± 0.49**	**2.54 ± 0.51**	**0.61**	**0.965**	**0.735**
**Grade I & II embryos/transferred embryos**	**146/190 (76.84%)**	**103/129 (79.84%)**	**50/66 (75.76%)**	**0.582**	**0.867**	**0.582**
**Implantation rate**	**36/190 (18.95%)**	**20/129 (15.5%)**	**5/66 (7.58%)**	**0.457**	**0.032**	**0.173**
**Clinical pregnancy**	**32/75 (42.67%)**	**17/50 (34%)**	**5/26 (19.23%)**	**0.356**	**0.036**	**0.286**
**Ongoing pregnancy**	**29/75 (38.67%)**	**15/50 (30%)**	**4/26 (15.38%)**	**0.346**	**0.031**	**0.264**

Values are expressed as n/n (%) unless otherwise indicated, G = group.

Table 4 shows the reproductive outcomes per transfer cycle in the salpingectomy group and the subgroups of the aspiration group. The implantation, clinical pregnancy and ongoing pregnancy rates were significantly higher in the salpingectomy group compared with the subgroup of patients with rapid re-accumulation of hydrosalpinx fluid (18.95% vs. 7.58%, 42.67% vs. 19.23% and 38.67% vs. 15.38%, respectively). No significant differences in the implantation, clinical pregnancy and ongoing pregnancy rates were detected between the salpingectomy group and the subgroup of patients with no re-accumulation of hydrosalpinx fluid (18.95% vs. 15.5%, 42.67% vs. 34% and 38.67% vs. 30%, respectively) or between the subgroup of patients with no re-accumulation of hydrosalpinx fluid and the subgroup of patients with rapid re-accumulation of hydrosalpinx fluid (15.5% vs. 7.58%, 34% vs. 19.23% and 30% vs. 15.38%, respectively).

## Discussion

In the present study, although the clinical pregnancy rate per started cycle and implantation rate were higher in the salpingectomy group compared with the aspiration group (40% vs. 27.5% and 18.95% vs. 12.82%, respectively), these differences failed to reach a statistical significance. The study sample size was not large enough to detect significant differences in implantation and pregnancy rates between both management options. In order to detect 12.5% difference in clinical pregnancy rate between salpingectomy group and aspiration group (i.e. 40% vs. 27.5%), each group should include 224 patients to give the study 80% power at a significance level of 0.05. Moreover, the results of our study suggested that rapid re-accumulation of the hydrosalpinx fluid (which occurred in 34.21% of patients who underwent aspiration) has a negative impact on implantation and pregnancy rates.

Meanwhile, the published studies have consistently revealed that salpingectomy improves the outcomes of IVF-ET [9], the studies comparing ultrasound guided aspiration with no intervention prior to IVF-ET have inconclusive results. In a retrospective study, Sowter et al, concluded that the drainage of hydrosalpinx fluid had no beneficial effect on the outcomes of IVF-ET [15]. On the other hand, a small retrospective study revealed that the aspiration of hydrosalpinx fluid improved the outcomes of IVF-ET [16].

In a randomized controlled trial, Hammadieh et al compared ultrasound guided aspiration of hydrosalpinx fluid with no intervention in a series of 66 patients with ultrasound visible hydrosalpinx undergoing IVF-ET [10]. The study was underpowered to examine various study endpoints because the study was terminated before targeted sample size (158 cases) was reached. In spite of this fact, the chemical pregnancy rate was higher in the aspiration group (43.8% vs. 20.6%, P value =0.04) and the clinical pregnancy rate was non-significantly higher in the aspiration group (31.3% vs. 17.6%, P = 0.20). Rapid re-accumulation of hydrosalpinx fluid occurred in 30.8% of patients in the aspiration group, the clinical pregnancy rate was 25% in patients with rapid re-accumulation of hydrosalpinx fluid and 33.33% in patients with no re-accumulation of hydrosalpinx fluid. Recently, in a randomized controlled trial, Fouda and Sayed compared ultrasound guided aspiration with no intervention in a series of 110 patients with ultrasound visible hydrosalpinx undergoing IVF-ET [11]. They reported that the implantation, clinical and ongoing pregnancy rates were significantly higher in the aspiration group (18.70% vs. 8.33%, 31.48% vs. 13.21% and 27.78% vs. 9.43%, respectively). Among the 54 patients who underwent aspiration of hydrosalpinx fluid, 10 patients had rapid re-accumulation of hydrosalpinx fluid, 2 patients had rapid re-accumulation of hydrosalpinx fluid and uterine fluid collection at the time of embryo transfer and 2 patients had uterine fluid collection at the time of embryo transfer. No pregnancies occurred in the 4 patients with uterine fluid collection. The implantation, clinical pregnancy and ongoing pregnancy rates were higher in the subgroup of patients with no re-accumulation of hydrosalpinx fluid compared with the subgroup of patients with rapid re-accumulation of hydrosalpinx fluid (21.98% vs. 13.64%, 37.5% vs. 20% and 32.5% vs. 20%, respectively) but these differences failed to reach statistical significance.

In the present study, no pregnancies occurred in the 3 patients who had re-accumulation of hydrosalpinx fluid and uterine fluid collection on the day of embryo transfer. The results of our study are in agreement with several studies which revealed that hydrosalpinx patients with uterine fluid collection at the time of embryo transfer have the worst reproductive outcomes [17,18]. Akman et al reported that 19.05% (4/21) of patients with ultrasound visible hydrosalpinx had uterine fluid collection [19]. Moreover, Chien et al reported that 5.63% (8/142) of patients with hydrosalpinx had uterine fluid collection at the time embryo transfer [20]. In both studies, none of the patients with uterine fluid collection conceived [19,20]. Sharara and McClamrock suggested that cryopreservation of embryos and subsequent transfer after salpingectomy is the best management option for this subgroup of patients [17].

The data presented in this study revealed that the aspiration of hydrosalpinx is less beneficial for the subgroup of patients with rapid re-accumulation of hydrosalpinx fluid and non-beneficial for the subgroup of patients with uterine fluid collection. It seems that the aspiration of hydrosalpinx fluid at the time oocyte retrieval prevents the efflux of hydrosalpinx fluid into the uterine cavity and therefore creates a non-hostile endometrium during the implantation period. Re-accumulation of hydrosalpinx fluid during the implantation period (i.e. first 2 weeks after embryo transfer) precludes any beneficial effect of aspiration. We think that salpingectomy, proximal tubal occlusion and hysteroscopic tubal occlusion by Essure micro-inserts are the best management options for the subgroups of patients with rapid re-accumulation of hydrosalpinx fluid or uterine fluid collection.

Several authors proposed combining ultrasound guided aspiration of hydrosalpinx with injection of sclerosing agent in situ to prevent rapid re-accumulation of hydrosalpinx fluid. In a retrospective study, Na et al compared ultrasound guided aspiration of hydrosalpinx fluid and sclerotherapy with salpingectomy in the management of patients with hydrosalpinx undergoing IVF-ET [21]. Forty one patients underwent salpingectomy before IVF-ET and 56 patients underwent aspiration of hydrosalpinx fluid and sclerotherapy with 95% ethanol and 5% tetracycline before the IVF cycle. In the sclerotherapy group, ultrasound examination was repeated after 2 weeks to detect re-accumulation of hydrosalpinx fluid. Sclerotherapy was repeated in 15 patients who had re-accumulation of hydrosalpinx fluid. The clinical pregnancy rate was comparable between both groups (38% vs. 40%). In a prospective study, 33 patients with ultrasound visible hydrosalpinx underwent ultrasound guided aspiration of hydrosalpinx fluid and sclerotherapy with 98% ethanol before IVF cycle and 19 patients with ultrasound visible hydrosalpinx underwent IVF-ET without any prior intervention. The implantation and clinical pregnancy rates were significantly higher in patients who underwent interventional ultrasound sclerotherapy (25% vs. 1.9% and 42.9% vs. 4.5%, respectively) [22].

For decades percutaneous ethanol injection was used in the treatment of unresectable early stage hepatocellular carcinoma and thyroid diseases [23]. Although percutaneous ethanol injection is generally safe, major complications as intraperitoneal hemorrhage, perforation of abdominal organs and shock have been reported. Moreover, infectious complications and abscesses formation had been reported in up to 1.5% of percutaneous ethanol injection sessions [24]. It was postulated that bacterial contamination of the necrotic tissues caused by ethanol injection was the main reason of infectious complications [23,24]. Both studies describing interventional ultrasound sclerotherapy of hydrosalpinx revealed that the main side effect was abdominal pain which was caused by leakage of alcohol into the abdominal cavity. There was no reported infectious morbidity or major complications [21,22]. Further larger well designed randomized controlled trials are needed to evaluate the efficacy and safety of interventional ultrasound sclerotherapy of hydrosalpinx.

The small sample size was the main limitation of our study. However, to the best of our knowledge this is the first study comparing salpingectomy with ultrasound guided aspiration of hydrosalpinx fluid in the management of patients with hydrosalpinx undergoing IVF-ET. Moreover, it is the largest study evaluating the effect of aspiration of hydrosalpinx fluid on the outcomes of IVF-ET.

## Conclusion

The small sample size could be the cause of failure of detecting significant increase in implantation and pregnancy rates in the salpingectomy group compared with the aspiration group. Further larger randomized controlled trials are needed to determine whether salpingectomy is more effective than aspiration of hydrosalpinx fluid or not. Moreover, the data presented in this study suggested that rapid re-accumulation of hydrosalpinx fluid is an obstacle against successful implantation and the cause of lower success rate with ultrasound guided aspiration of hydrosalpinx fluid compared with salpingectomy.

## Abbreviations

BMI: Body mass index
IVF-ET: In vitro fertilization embryo transfer
PCOS: Polycystic ovary syndrome
FSH: Follicle stimulating hormone
E2: Estradiol
HCG: Human chorionic gonadotropin
2PN: 2 pro-nucleate embryos
HOXA10: Homeobox A10
HP-uFSH: highly purified urinary follicle stimulating hormone
